# Increased temperature delays the late-season phenology of multivoltine insect

**DOI:** 10.1038/srep38022

**Published:** 2016-12-01

**Authors:** Adam Glazaczow, David Orwin, Michał Bogdziewicz

**Affiliations:** 1 Department of Systematic Zoology, Adam Mickiewicz University, Umultowska 89, 61-614 Poznan, Poland; 2 University of Bedfordshire, Park Square, Luton, Bedfordshire LU1 3JU, UK

## Abstract

We analyzed the impact of increased water temperature on the late-season phenology of the mayfly (*Baetis liebenauae*). The River Gwda, unlike two other examined rivers (controls), has reservoirs along its length and thus, higher water temperature. Elevated water temperature prolonged summer diapause of the mayfly and shifted its life cycle to the later autumn: the last generation of mayflies started development later in the Gwda than in the control rivers. This translated into terrestrial stages (subimagos) of the insect being more abundant at the water surface in the late autumn in the Gwda river than in the control rivers. The low water temperature in the late autumn hampers subimagos emergence from the water surface. Thus, the altered insect phenology at Gwda resulted in a largely lost generation. However, the effect of reservoirs on the river water temperature was context-dependent, with the heating effect (and the impact on mayfly phenology) weaker in the year with lower average air temperature. In summary, warming blurred the environmental cue used by mayflies to tune their phenology, which resulted in a developmental trap. Since the projections of increases in global temperatures reach even 6.4 °C, reported mechanisms will potentially also occur in non-transformed watercourses.

Phenology is the timing of seasonal activities of plants and animals such as flowering or mating. Alterations in phenology are among the best-supported effects of climate change on organisms^1^,^2^,^3^,^4^,^5^. So far, however, most studies focus on early-season phenology such as the earlier start of reproduction, flowering or arrival of migrant birds^1^,^4^,^6^,^7^,^8^. Alterations in late-season phenology are less understood but these studies will enable better understanding of population consequences of these shifts, especially in insects^1^,^6^,^9^,^10^.

In ectotherms, increased ambient temperature has direct consequences for metabolic rates, activity patterns, and developmental rates^11^,^12^,^13^,^14^,^15^,^16^. Multi-generation (i.e. multivoltine) insects are believed to take advantage of climate warming because of the prolongation of the time for development due to an earlier start in spring^11^,^17^. The accelerated early-season phenology results in the increase in the number of generations per year that could boost the population growth^11^. Recently, however, it has been hypothesized that insects could be negatively affected by such phenomena if the decision to add another generation instead of entering a diapause is maladaptive^6^. The crucial aspect is whether the development of the added generation is completed before the season is finished (‘the lost generation hypothesis’^6^). In a similar manner, altered water temperature could be detrimental to insects if it prolongs the summer diapause which results in the autumn generation beginning its development too late in the season. Multivoltine species, whose development is temperature-dependent, might be especially susceptible to such developmental traps^6^.

Mayflies (Ephemeroptera) are insects with numerous multivoltine species, and the number of generations within life cycles could vary among populations of the same species^18^,^19^. The development of both eggs and larvae is temperature-dependent^20^,^21^,^22^. On one hand, an increase in the water temperature accelerates mayfly development, which could potentially translate into an increase in the number of generations per year^22^,^23^,^24^,^25^. On the other hand, the duration of the diapause might increase when temperature increases because periods of chilling could be essential for diapause termination^15^,^26^,^27^. Thus, increased water temperature may potentially prolong summer diapause. Both mechanisms (added generation or prolonged diapause) will result in emergence of the last-season generation of mayflies delayed for later in the season. At the same time, the emergence of mayfly subimagos from the water surface for moulting and mating is likely to be determined by the water temperature^28^,^29^. If the emergence from the water surface is negatively affected by temperature, low late-season temperatures could prevent it and harm mayfly populations (the “lost generation”).

We used water reservoirs and populations of the mayfly *Baetis liebenauae* as our model system and tested the ‘lost generation’ hypothesis. Hydroelectric power plants require dams built along rivers to stack water creating reservoirs that alter the water temperature of rivers. Such reservoir-induced alterations in temperature have a marked effect on macroinvertebrate communities^24^,^30^,^31^,^32^,^33^. The increased temperature could be used to experimentally evaluate the impact of warming on insect phenology^24^,^34^. Generally, the water in shallow reservoirs heats up more quickly than the river current in summer and cools down more rapidly in colder months^35^. Thus, water discharge from reservoirs increases the river water temperature during summer and decreases it during autumn^35^.

The range of *Baetis liebenauae* reaches Finland to the north and France and Italy to the south^36^. Over most of its range it is considered a bivoltine species that overwinters in the egg stage^37^. A polivoltine cycle is less common, although in Italy, in stable water temperatures (12–17 °C), three generations of *B. liebenauae* during one year have been observed^38^. Emergence of subimago and mating flights of imagoes occur between May – June (spring generation) and August – September (autumn/last generation). Summer diapause occurs in July and is induced and terminated by temperature^39^. *Baetis liebenauae* occurs in the unpolluted rivers of the medium size, larvae occupy patches of aquatic vegetation in the main current of the river^39^ (for more detailed information about the habitat preferences and life cycle see Głazaczow^39^).

Our objective was to test the following predictions. (1) Water discharge from reservoirs increases river temperature in summer. This in turn, (2) shifts life cycle and emergence of the mayfly (*Baetis liebenauae*) to later in the autumn (i.e. induces temporal delay). (3) The late emergence follows from delayed induction of development of the last generation in the heated river. Finally, the (4) shift of the cycle translates into reduced success of emergence of the mayfly winged stage because the emergence success is determined by the water temperature.

## Results

### Prediction 1: water discharge from reservoirs increases river temperature in summer

**Figure 1:**
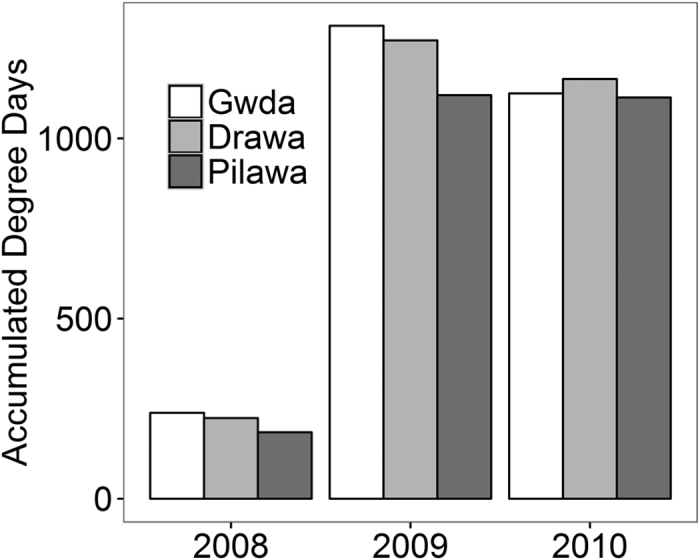
Accumulated degree days in three studied rivers. Average mean daily temperatures are accumulated for the 1^st^ August – 31^st^ October. Gwda has dams built along its course to stack water which created reservoirs. Note that 2008 could not be directly compared to the other years because temperature was recorded on a limited number of days (loggers malfunction, see Methods for details).

The average daily water temperature ranged from 13.6–21 °C in August, with values exceeding 20 °C recorded only in the River Gwda (Supplementary Table 1S). Temperature declined to 0.1–3 °C in the late autumn (November). Generally, the River Gwda was warmer than the other rivers during the warmer part of the year (August/September) by 0.5–2 °C and cooler by the same values during the colder period (October/November), although the difference was smaller in generally colder year 2010 (Table 1S). Similarly, accumulated degree days (accumulated mean temperature per day for the August – October period) were higher at Gwda than at the two other rivers in 2008 and 2009 but not in 2010 (Fig. 1).

### Prediction 2: increase in temperature shifts life cycle and emergence of the mayfly to later in autumn

**Figure 2:**
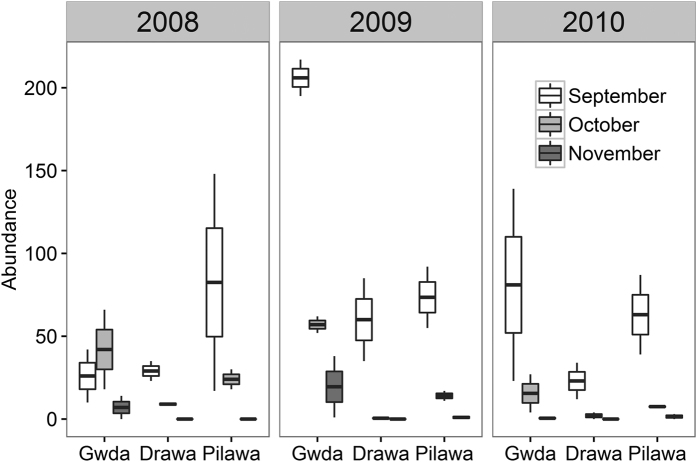
Abundance of mayfly larvae in the three studied rivers in September, October, and November in 2008–2010. Water temperature in the Gwda is elevated by reservoirs (in 2008 and 2009, see Methods). Boxes denote 25th, 50th, and 7 h percentiles; whiskers represent the lowest and highest datum within the 1.5 interquartile range of the lower and upper quartile.

The larval abundance was lowest in the Drawa (Drawa vs. Gwda: χ^2^ = 403.15, *p* < 0.001; Drawa vs. Pilawa: χ^2^ = 107.98, *p* < 0.001, N = 54) and highest in the Pilawa (Pilawa vs. Gwda: χ^2^ = 98.58, *p* < 0.001; Fig. 2), although the numbers varied among years (χ^2^ = 227.92, p < 0.001). In accordance with our prediction, the numbers of recorded mayflies in the Gwda in October and November were higher than in the other two rivers in all years, although the differences in November 2010 were not significant (Fig. 2, Supplementary Table 2S).

### Prediction 3: The late emergence follows from delayed induction of development of the last generation in the heated river

**Figure 3:**
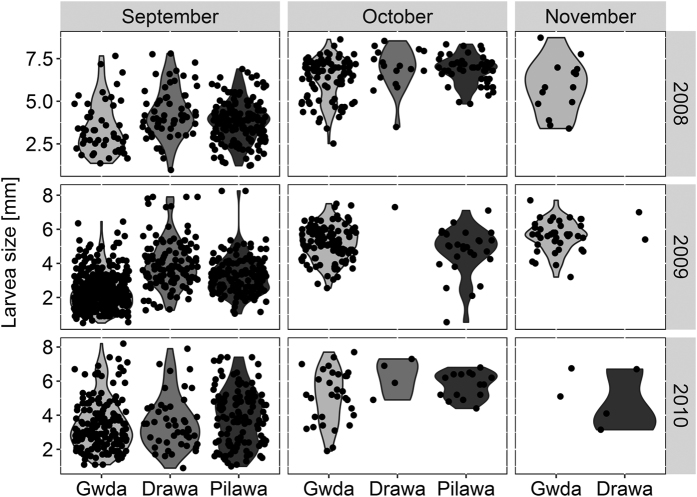
Mayfly larval size in the three studied rivers in September, October, and November in 2008–2010. Water temperature in the Gwda is elevated by reservoirs (see Methods). The width of violin at each point represents the density of observations near the particular value.

We used larval size as a surrogate for the stage of their development^23^,^25^,^34^. In accordance with our hypothesis, larvae were smaller in the Gwda than in the other two rivers in September (2008: χ^2^ = 15.45, df = 2, *p* < 0.001; 2009: χ^2^ = 227.47, df = 2, *p* < 0.001, N = 1695; Fig. 3), although the difference in 2010 was only marginally significant (χ^2^ = 5.76, df = 2, *p* = 0.056). The difference in larval size among rivers became smaller in October (in comparison to September: river × month:χ^2^ = 9.50, df = 2, *p* = 0.008). In November the larvae were almost exclusively present in the Gwda. Larval size in the Gwda did not differ between October and November (χ^2^ = 0.07, *p* = 0.78).

### Prediction 4: The shift of the cycle translates into reduced success of emergence of mayfly winged stage

**Figure 4:**
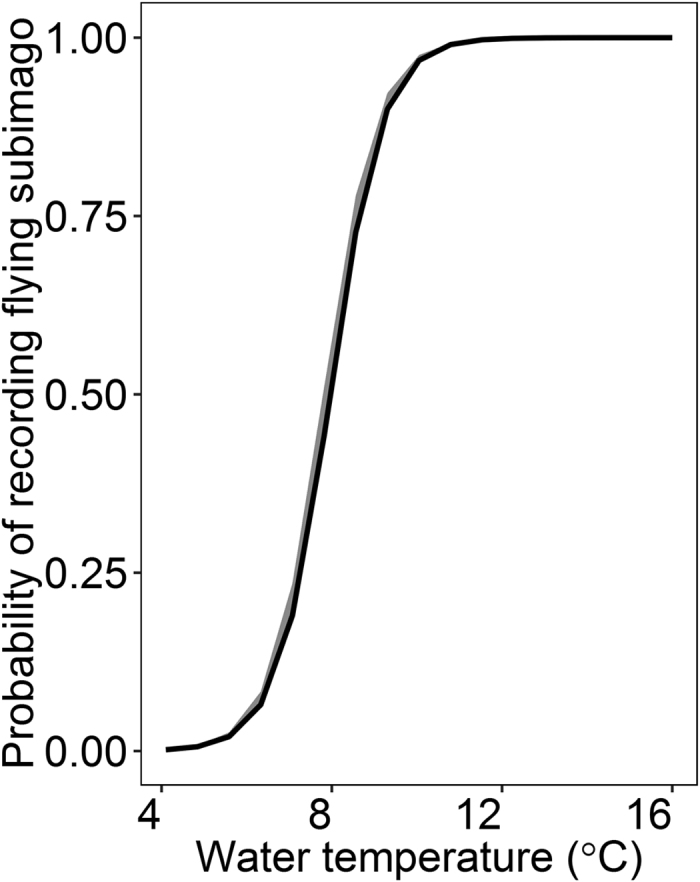
The relationship between water temperature and the probability of mayfly emergence. Estimates are derived from the GLM with the water temperature and river used as explanatory variables (see Methods for details). Shaded regions indicate 95% confidence intervals.

As predicted, the temperature had a strong positive impact on the probability of mayfly emergence (i.e. the probability that the individual is observed flying, χ^2^ = 2180.85, *p* < 0.001, N = 2511; Fig. 4). When the temperature was above 10–12 °C (i.e. the mean water temperature recorded in September, see Table 1S) mayflies readily emerge from the water surface. However, the probability declined gradually when the temperature dropped below 10 °C (i.e. temperature in October, see Table 1S). When the temperature reached about 5 °C (i.e. temperature in November, Table 1S) all subimagos stayed on the water surface.

**Figure 5:**
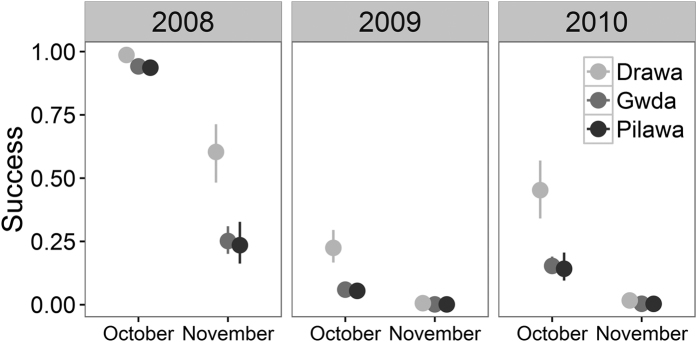
The influence of the phenology on the emergence success of mayflies. Water temperature in the Gwda is elevated by reservoirs (see Methods). Success is defined as the proportion of individuals that managed to take-off from the water surface. Estimates are derived from the GLM with the river, month, year and the three-way interaction term used as explanatory variables (see Methods for details). September is not shown on the figure because the success was 99–100% in all the rivers. Whiskers indicate standard errors.

As a result of the strong relationship between water temperature and emergence, the overall emergence success differed between months (χ^2^ = 809.16, df = 2, *p* < 0.001, N = 2511) and rivers (χ^2^ = 12.35, *p* = 0.002; Fig. 5). The impact of the phenology on emergence success did not differ between rivers (river × month: χ^2^ = 3.58, df = 3, *p* = 0.28). The emergence success was highest in September (September vs. October: χ^2^ = 567.75, *p* < 0.001; September vs. November: χ^2^ = 169.07, *p* < 0.001) and lowest in November (χ^2^ = 898.38, *p* < 0.001).

## Discussion

Our results support the lost generation hypothesis^6^. Water reservoirs built along the river altered water temperature. Increased temperature in summer delayed the emergence of the last generation of mayfly for colder months (October and November). Since the success of the insect emergence was positively related to the water temperature, it was extremely low in that period (Fig. 5). Thus, the mayflies present in large numbers in the Gwda were unable to take-off for moulting and mating.

The higher abundance of mayflies in October and November in the Gwda could be simply the result of higher abundance of mayflies in this river in September. However, this was not the case since in 2008 the insect abundance in the Gwda was even higher in October than in September (Fig. 2), indicating delayed phenology. Moreover, in 2009 the decrease in larval numbers was stronger in the Drawa and Pilawa than in the Gwda (*p* < 0.05). This indicates that a higher proportion of individuals stayed in the water in the Gwda. On the other hand, in 2010 the decline in numbers was similar between the rivers (*p* < 0.05), indicating negligible or no phenological shift that year.

In 2010, mayflies in the three studied rivers exhibited negligible differences in development phenology. This appeared to result from the differences in the ambient temperatures between studied years (Fig. 1, Table 1S). Based on our data, we speculate that in the warm years (2008 and 2009) the overall higher air temperatures allowed the reservoirs to heat up in summer months, which resulted in higher water temperature in the Gwda. This translated into the later onset of development of the last generation of mayflies in the Gwda (Fig. 4, upper and mid left box). Thus, a high proportion of mayflies was still present at the water surface in October and November (Fig. 2). In contrast, in the colder 2010, the reservoirs were not efficient at heating up the river water: the difference in water temperatures between the rivers was lower (Fig. 1, Table 1S). As a result, larvae in all three rivers started to develop over a similar time (Fig. 3. bottom left box) and the late-season phenology of mayflies at Gwda was not altered (Fig. 2).

The context-dependence of the impact of reservoirs on mayflies could be of potential significance for the insect population dynamics. Our results suggest that the negative impact of the reservoir-induced altered water temperatures on mayflies is relaxed in colder years which could preserve local populations. However, ambient temperatures are increasing globally^40^, and this trend is especially strong in Central Europe^1^. Thus, the frequency of such cold years is likely to decrease. This could have detrimental effects on populations of multivoltine insects in rivers with reservoirs. Moreover, in the warmer periods the temperature of water in rivers could rise to insect-threatening levels even without manmade reservoirs.

The delayed onset of development of the last generation of mayflies could be caused by two opposite mechanisms: added generation or prolonged diapause. The influence of temperature on insect development is generally species-specific^26^,^27^,^41^. On one hand, the temperature could alter the characteristics of the diapause, e.g. by decreasing its duration^15^,^26^. This, together with accelerated development could increase the number of generations within one season. On the other hand, higher temperatures might postpone diapause termination which would lead to a later onset of the development of the last generation^15^,^34^. Indeed, the emergence of a second generation of the mayflies is delayed by the later river cooling^34^. Given the magnitude of the difference in phenology between heated and control rivers in our study, and our experience in working with that species, we believe that the latter is the case. Nevertheless, recognition of whether the mechanism consisted of an increase in number of generations or a prolonged diapause could be a valuable venue for future research.

Regardless of the mechanism, whether the altered phenology is positive, neutral or detrimental for mayfly populations depends on whether the last generation will finish its development before the end of the season^6^. Our results show that the increased water temperature is likely to be detrimental to mayflies because the postponed late-season phenology resulted in the suicidal last generation^6^,^42^. On the other hand, some portion of individuals finished development and survive, providing potential for evolutionary response of the population to altered temperature regimes. Long-term studies focused on population dynamics that would include tracking whole life cycles are needed to evaluate the impact of altered phenology on insect populations. Moreover, we cannot rule out that the found effect was caused by some other, unmeasured differences among studied rivers. Thus, further studies based on larger number of reservoirs would be a valuable area of future investigations.

It is critical for fitness that the phase of reproduction is synchronized with favorable seasons, while diapause with unfavorable periods^11^,^43^. We found that increased temperature leads to the later onset of development of the last generation of mayflies. Thus, numerous individuals in the Gwda river failed to emerge and remained in cold water outside of a diapause, before the cold-tolerance mechanisms had been established^10^,^15^,^44^. The changed temperature distorted the quality of information given by environmental cues that insects use to make developmental decisions^6^. This led to the developmental trap, a special case of evolutionary trap^6^,^45^. The estimates of global temperature increase oscillate between 1.1 and 6.4 °C by year 2100 (IPPC, 2013), which makes mechanisms reported here potentially relevant even for non-altered watercourses.

## Methods

### Study site

**Figure 6:**
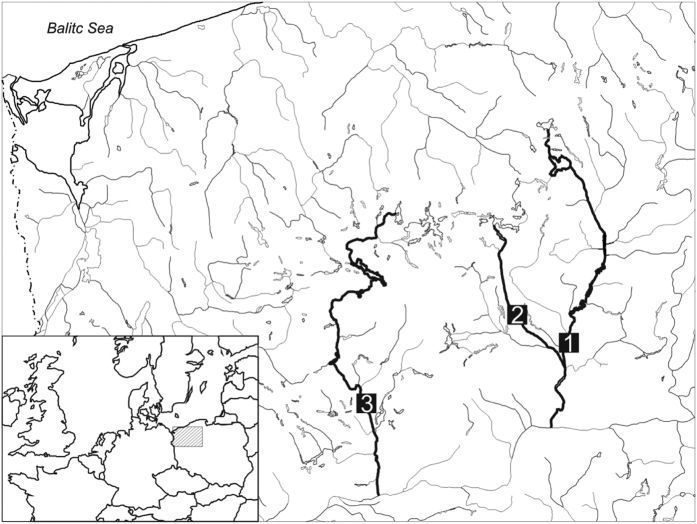
Study area. Tags indicate the sampling sites on the rivers: 1 – Gwda, 2 – Pilawa, 3 – Drawa. The map was created using the CorelDRAW Graphics Suite 12 (http://www.coreldraw.com/).

We selected three Pomeranian rivers (Fig. 6) from the catchment of North West Poland to investigate the impact of increased water temperature on the life cycle of *Baetis liebenauae*. The main sampling site (Fig. 6; site 1) was located on the River Gwda, below four water reservoirs. The study site is located over 10 km from the nearest dam which precludes the direct effects of water discharge on the focal insect abundance (Głazaczow, personal observation). As control sites, we selected two unregulated rivers which flow through a similar landscape: the Pilawa (site 2), and the Drawa (site 3).

The middle section of the Gwda is divided into riffles and pools and is overgrown mostly by the fennel-leaved pondweed (*Potamogeton pectinatus*). Steep gradient and diversified substratum makes the water well aerated. The second site (Pilawa) is the biggest tributary of the River Gwda. The bed consists of the sand and gravel that covers the tills. The third site (Drawa) flows parallel to the Gwda and has similar hydromorphological parameters to the two other rivers (Table 1).

We measured the river’s water and air temperatures from August to November (2008–2010) using i-Button temperature loggers (one logger per study site, temperature range: −5° to +26 °C, resolution 0.125^o^C). We placed the loggers ca. 1 meter from the riverbank to ensure that the loggers are submerged under the water throughout the study period. At each site the logger was placed in similar microhabitat (i.e. no vegetation). We took six temperature records during the day (every 4 hours) to measure the daily regime of water temperature. The temperature in August and September 2008 was recorded manually because of loggers malfunction.

We assessed mayfly abundance on the basis of quantitative samples collected by Surber nets (mesh 0.25 mm) from 0.1 m^2^. Each month (September to November, 2008–2010) we collected two samples from each river. Since larvae of the focal species live on the aquatic plants, we placed the net at the edge of the vegetation patch, we cut the vegetation and collected it. Material was transported to the laboratory and rinsed. Each time the collected vegetation was dominated by the fennel-leaved pondweed. We counted mayfly individuals in the laboratory and measured them to the nearest 0.1 mm.

Moreover, we observed and counted the winged stage (subimagos) of the mayfly along two 50 m^2^ transects (each lasted 15 minutes). We distinguished two types of mayfly behaviour: (1) flying (i.e. individuals that were flying over the water), and (2) floundering or skipping (i.e. individuals that tried to escape from the water surface but moved with the current down-stream). We identified emerging mayflies to the species by collecting them with the emergence traps. In September more than 90% of all trapped individuals were *B. liebenauae*. In all other months we captured only *B. liebenauae* (Glazaczow, unpublished data).

### Statistical analysis

We analyzed the data using Generalized Linear Models (GLMs) and Generalized Linear Mixed Models (GLMMs) implemented via “lme4” package (Bates *et al*. 2011) in R software (R Development Core Team 2014). We tested for statistical significance of fixed factors with marginal type II likelihood ratio tests, implemented by the ‘car’ package^46^. Unless stated otherwise, number of degrees of freedom for fixed effects equals 1. All models with three-way interactions include all nested two-way interactions. We ran pairwise comparisons between different levels of fixed effects by sub-setting the dataset and re-fitting focal models. In models testing for differences among rivers we decided to use ‘river’ (three levels) instead of ‘treatment’ (two levels) as fixed effect because we a-priori expected substantial differences among rivers. Thus, we have used a more conservative approach and did not pool Pilawa and Drawa into one category.

### Prediction 1: water discharge from reservoirs increases river temperature in summer

We tested the differences in water temperature among the rivers using a GLMM with the water temperature as the response variable, river, month, year, and the three-way interaction term river × month × year as the fixed factors. To account for repeated measures we included sampling day as the random effect. We used identity-link function and assumed the Gaussian error distribution term.

### Prediction 2: increase in temperature shifts life cycle and emergence of the mayfly to later in the autumn

To test whether the abundance of mayflies is higher in the Gwda than in the control rivers late in the season (October and November), we built a GLM with mayfly count as response variable and river, month, year and three-way interaction term river × month × year as fixed effects. We used log-link function and assumed the Poisson error distribution term.

### Prediction 3: The late emergence follows from delayed induction of development of the last generation in the heated river

To test whether development of mayflies started later in the season in the Gwda we used larval size as a surrogate for the stage of their development. Such procedure is widely used in mayfly studies^23^,^25^,^34^,^47^. We built a GLM with larval size as response variable, river, month, year, and three-way interaction term between those variables as explanatory variables. We used identity-link function and assumed the Gaussian error distribution term.

### Prediction 4: The shift of the cycle translates into reduced success of emergence of the mayfly winged stage

We analyzed whether temperature and phenology influence the mayfly emergence success by building two binomial GLMs with logit-link function. In both models we used mayfly emergence (0/1) as the response variable (where 0 was floundering or skipping and 1 was flying). In the first model, we analyzed the influence of water temperature on the probability of mayfly emergence. Here, we used the water temperature as the explanatory variable and the river as a covariate. We excluded time variables (month and year) to avoid covariation with temperature.

In the second model, we tested whether delayed phenology affected emergence success of mayflies defined as the proportion of individuals that successfully emerged from the water surface. We used river, month, year and the interaction term between river and month as fixed effects.

## Additional Information

**How to cite this article**: Glazaczow, A. *et al*. Increased temperature delays the late-season phenology of multivoltine insect. *Sci. Rep.* **6**, 38022; doi: 10.1038/srep38022 (2016).

**Publisher's note:** Springer Nature remains neutral with regard to jurisdictional claims in published maps and institutional affiliations.

## Supplementary Material

Supplementary Materials

## Figures and Tables

**Table 1:** Main characteristics of rivers Gwda, Drawa and Pilawa.

River	Gwda	Drawa	Pilawa
Catchment [km^2^]	4,947	3,296	1,352
Length [km]	145	186	82
Width* [m]	30	25	15
Depth* [m]	0.8	0.9	0.6
Slope [‰]	0.8	0.6	0.4
Current* [m/sec]	0.5	0.7	0.6
Discharge [m^3^/sec]	27	21	8

Asterisks* indicate max values.
