# Supplementary material for: Increased temperature delays the late-season phenology of multivoltine insect
Source: Sci Rep. 2016 Dec 1;6:38022. doi: 10.1038/srep38022 (PMC5131318; doi:10.1038/srep38022)
Supplement: Supplementary Materials [file srep38022-s1.pdf]

Galazaczow A., Orwin D., Bogdziewicz M. Increased temperature delays the late-season phenology of multivoltine insect. Scientific Reports

Table 1S. Average air and water temperatures during the course of the study. Statistics and *p*-values are given for the comparison between the Gwda (river with reservoirs) and two control rivers. Temperature in August and September 2008 lack full statistics because of i-loggers malfunction (see Methods for details).

	Air Temperaure	SD		August			September			October			November		
				Average	SD	t statistics, *p*-value	Average	SD	t statistics, *p*-value	Average	SD	t statistics, *p*-value	Average	SD	t statistics, *p*-value
2008	10.50	5.2	Gwda	18.80	1.40		12.85	0.07		8.70	0.89		4.63	2.08	
			Drawa	17	-		12.90	-		9.34	0.89	-2.74, p = 0.006	5.33	2.10	-8.52, p < 0.001
			Pilawa	16.9	-		12.0	-		8.60	1.18	6.64, p < 0.001	4.25	2.24	15.23, p < 0.001
2009	10.50	6.56	Gwda	18.71	0.87		15.18	1.40		8.11	1.94		4.99	0.86	
			Drawa	18.01	0.84	- 9.79, p < 0.001	14.80	1.31	-5.18, p < 0.001	8.69	1.67	8.13, p < 0.001	5.57	0.66	7.92, p <0.001
			Pilawa	17.69	1.15	-14.35, p < 0.001	13.07	2.49	-28.98, p < 0.001	5.77	1.63	-32.97, p < 0.001	6.49	0.97	11.75, p <0.001
2010	8.25	6.02	Gwda	18.08	1.39		12.68	0.75		7.08	1.91		4.96	1.58	
			Drawa	18.06	1.40	-0.36, p = 0.71	13.07	0.70	10.51, p < 0.001	7.98	1.85	24.67, p < 0.001	5.40	1.68	11.67, p <0.001
			Pilawa	17.67	1.70	-10,64, p <0.001	12.63	0.84	-1.32, p = 0.18	7.14	1.74	1.45, p = 0.14	5.10	1.77	3.67, p <0.001

Table 2S. Results of post-hoc tests of differences in abundance of mayfly larvae among the studied rivers. In all tests the degrees of freedom equals 1. Values given in italics are statistically non-significant at the *p* level 0.05. In all comparisons numbers in the Gwda are higher than in the other rivers.

	**Gwda vs. Drawa**		**Gwda vs. Pilawa**		**Drawa vs. Pilawa**	
	October	November	October	November	October	November
**2008**	$\chi^2 = 107.98$	$\chi^2 = 19.40$	$\chi^2 = 9.94$	$\chi^2 = 19.40$	$\chi^2 = 14.15$	N.A.
**2009**	$\chi^2 = 147.94$	$\chi^2 = 54.06$	$\chi^2 = 55.85$	$\chi^2 = 40.85$	$\chi^2 = 31.50$	*$\chi^2 = 2.77$*
**2010**	$\chi^2 = 23.64$	*$\chi^2 = 1.38$*	$\chi^2 = 5.67$	*$\chi^2 = 1.04$*	$\chi^2 = 6.78$	N. A.
